# Oral health knowledge of health care workers in special children’s center

**DOI:** 10.12669/pjms.311.6477

**Published:** 2015

**Authors:** Amjad Wyne, Nouf Hammad, Christian Splieth

**Affiliations:** 1 Amjad Wyne, MDS. Professor, Department of Pediatric Dentistry and Orthodontics, King Saud University College of Dentistry, Riyadh, Saudi Arabia.; 2 Nouf Hammad, MSc. Associate Professor, Department of Pediatric Dentistry and Orthodontics, King Saud University College of Dentistry, Riyadh, Saudi Arabia.; 3 Christian Splieth, PhD. Professor, Preventive and Pediatric Dentistry, University of Greifswald, Rotgerberstr, Germany.

**Keywords:** Oral health knowledge, Special health workers, Special children

## Abstract

**Objective:**

To determine the oral health knowledge of health care workers in special children’s center.

**Methods:**

A self-administered questionnaire was used to collect following information: demographics, oral hygiene practices, importance of fluoride, dental visits, cause of tooth decay, gingival health, and sources of oral health information. The study was conducted at Riyadh Center for Special Children in Riyadh City from December 2013 to May 2014.

**Results:**

All 60 health care workers in the center completed the questionnaire. A great majority (95%) of the workers brushed their teeth twice or more daily. More than two-third (71.7%) of the workers knew that fluoride helps in caries prevention. One in five (21.7%) workers thought that a dental visit only becomes necessary in case of a dental problem. Similarly, 13.3% of the workers thought to “wait till there is some pain in case of a dental cavity” before seeking dental treatment. The workers ranked soft drinks/soda (98.3%), flavored fizzy drinks (60%) and sweetened/flavored milks (43.3%) as top three cariogenic drinks. A great majority (95%) of the workers correctly responded that blood on toothbrush most probably is a sign of “gum disease”. Dentists (50%) and media (45%) were the main source of their oral health information. There was no significant difference (*p* > 0.05) in workers’ response in relation to their specific job.

**Conclusion:**

The special health care workers in the disabled children’s center generally had satisfactory oral health knowledge and practices.

## INTRODUCTION

Maintaining optimal oral and dental health in intellectually and/or physically challenged children is of utmost importance, as these children usually suffer from several associated general health problems in addition to their primary condition. Poor dental health not only further compromises their general health but may also aggravate negative social interaction with these children.^1^ The special children need continuous dietary supervision and assistance in maintaining optimal oral hygiene due to their poor intellectual development and compromised neuromuscular coordination. It is important that health care workers in special children’s centers understand the importance of optimal oral health.^2^ It is contemplated that health care workers with good oral health knowledge and preventive practices would play a better role in maintaining optimal oral health in the special children under their care.^3^

A higher prevalence of dental diseases has been reported in children with disabilities in different parts of the world,^4^^-^^6^ including Arabian Gulf countries.^7^^-^^9^ Several studies have indicated towards a need for improved prevention of dental diseases in special children.^10^^,^^11^

Parents/care takers and health care workers of special children’s centers play a vital role in maintaining optimum oral health in these children.^12^ There have been some studies regarding oral health knowledge and attitude in parents of special children.^12^^-^^14^ Similarly, health care workers with good oral health knowledge and practices are likely to play a positive role in oral health care of special children.^3^ However, there are no such studies published on oral health knowledge of health care workers in special children’s centers. The information obtained through such studies would assist in oral care education of health care workers in special children’s centers, which consequently is likely to have a positive effect on the oral health of these children. The aim of the present study was to determine the oral health knowledge of health care workers in a special children’s center.

## METHODS

A self-administered questionnaire was developed for the study. The questionnaire was pre-tested; and appropriate changes were carried out to improve its comprehensibility for the health care workers. The following information was collected about oral health knowledge and practices in the special health care workers:

- Demographic information such as age, gender, and job title
- Importance of good dental health
- Importance of good dental health for general health
- Oral hygiene practices
- Importance and various sources of fluoride
- Frequency and reason for dental visits
- Possible causes of tooth decay
- Probable reason(s) for bleeding gums and action that should be taken
- Main source of oral health information

The study was registered with King Saud University College of Dentistry Research Center (CDRC), and ethical approval was obtained. The “Riyadh Center for Special Children” where the study was conducted from December 2013 to May 2014 is the main center for special children in Riyadh City where health care and education is provided to children with various conditions/disabilities (such as cerebral palsy and Down’s syndrome). The center was visited by one (NH) of the researchers. The questionnaires were handed over to the workers for completion. The questionnaires had a covering letter explaining the research objectives and ensuring anonymity/confidentiality of the information obtained through the questionnaire.

The data collected through the questionnaires were entered into the computer utilizing FOXPRO program. Statistical Package for Social Sciences (SPSS - Version #19) was utilized to drive various frequencies and data analyses. Chi-Square test was used to determine any significant (*p*≤ 0.05) difference in various responses in terms of specific job type of the workers. 

## RESULTS

All the 60 health care workers (58 females and two males) in the center completed the questionnaire. The mean age of the workers was 31.0 (SD 7.0) years ranging from 22 to 55 years. The sample consisted of 13 (21.7%) nursing staff, 19 (31.7%) physiotherapists/speech therapists and 28 (46.7%) special teachers.

The workers answers to questions about importance of oral health, frequency of dental visits and sources of oral health information are listed in Table-I. A great majority was aware of the importance of healthy teeth in relation to chewing (90%), esthetics (80%) and speech (68.3%). Similarly, almost all (95%) of the workers were aware of the importance of good dental health for optimal general health. Approximately three in every four (73.3%) workers knew that one should visit a dentist twice a year for regular check-ups; however, one in five (21.7%) workers thought that dental visit is only necessary in case one has a dental problem. Dentists (50%) and media (45%) were the main sources of oral health information for the workers.

Table-II presents the workers’ response to questions regarding their oral hygiene. A great majority (95%) of the workers was brushing either twice daily, three times per day or after each meal. Almost all (96.7%) the workers were using either toothbrush (71.7%) or combination of toothbrush and miswak (25%) for cleaning their own teeth. Miswak is a traditional wooden toothbrush used in many parts of the world especially in Arabia. Those who were using toothbrush were all using toothpaste with the brush.

All the workers except one had heard about fluoride (Table-III). Although 71.7% of the workers knew that fluoride helps preventing caries, 25% thought that its main benefit is to whiten the teeth (Table-III). Various sources of fluoride included; toothpastes (93.3%), direct application by dentist (51.7%) and drinking water (20%). 

Almost all the special health workers were aware that sugary foods mainly cause tooth decay. When asked about drinks that can have harmful effects on the teeth, the workers ranked soft drinks/soda (98.3%), flavored fizzy drinks (60%) and sweetened/flavored milks (43.3%) as top three in the list (Table-III). In response to the question about what action needs to be taken when one feels a cavity starting in a tooth; 73.3% correctly responded as “visit a dentist immediately”. However, 13.3% thought “to wait till there is some pain” and some (5%) preferred to “wait till it is large enough to be filled” .

A great majority (95%) of the workers correctly responded that blood on toothbrush during daily brushing most probably is a sign of gum disease (Table-IV). However, they differed in their opinion about what action to be taken on seeing the blood; with opinions ranging from seeing a dentist (55%), continue brushing (36.7%), stop brushing the area that is bleeding (6.7%) and stop brushing the teeth (1.7%).

As there were only two male workers in the center, gender-specific analysis was not feasible. A comparison of responses in relation to specific job of the workers did not show any meaningful differences (*p *> 0.05). 

## DISCUSSION

The present study has provided information about oral health knowledge and practices of special health care workers in a Disable Children’s Center. No similar study has been published in English language health literature. The information would help in assessment of oral health knowledge needs in these workers, and consequently designing educational programs for such workers.

The knowledge of the special care workers was generally satisfactory about the functions of healthy mouth and importance of good dental health for general health. However, one-fifth of them thought that dental visit is only necessary in case one has a dental problem. Some previous studies of health care workers have also reported a tendency towards being symptom-oriented in utilization of oral health services.^15^^,^^16^ Regular dental check-up visits are vitally important to prevent dental problems. These visits provide an opportunity for dentists to take clinical preventive measures such as dental prophylaxis, topical fluoride application and placement of fissure sealants (if necessary) and reinforce the healthy home dental care.

The workers’ knowledge about oral hygiene and practices was profound, which reflects positively on these workers. It has been reported that health care workers who themselves act on the advice they give, provide better counseling and motivation for their patients to adopt such health advice.^17^ About one-fourth of the workers were using combination of toothbrush and miswak (a wooden toothbrush), which could be considered as an additional healthy trend, in view of the oral health benefits associated with miswak.^18^

Though the workers’ knowledge about fluoride can be termed as satisfactory, only one in five workers recognized drinking water as source of fluoride. A systematic review of 214 studies concluded that water fluoridation was associated with an increased proportion of caries-free children and a reduction in the number of teeth affected by dental caries.^19^ Fluoridated water is also the most cost-effective source of fluoride and carries advantages such as independence from compliance and non-selective benefits for all, over other fluoride sources.^20^ Previous studies in parents of special children have also shown that very few parents were aware of water as possible source of fluorid.^21^

The workers’ knowledge about effect of sugary foods and sweetened/acidic drinks on teeth seems adequate; which should have positive effect on the knowledge of parents of the special children. There have been several previous reports about inadequate awareness in special children’s parents about harmful effects of soft drinks/soda, flavored fizzy drinks and sweetened/flavored milk on their children’s teeth.^13^^,^^21^ The information about possible harmfulness of sweetened and acidic drinks is very important in children with special care needs, as many of these children are exposed to additional caries risks such as compromised oral hygiene due to poor neuromuscular coordination, inadequate intellectual development, use of soft and sweetened foods and sweetened medicines.

**Table-I:** Response to questions regarding importance of oral health, frequency of dental visits and main source of oral health knowledge

*Question*	*Number*	*%*
What is the importance of good dental health? (multiple responses possible)		
1. Effective Chewing	54	90.0
2. Speech	41	68.3
3. Esthetics	48	80.0
4. Not so important	1	1.7
Is good dental health important for optimum general health?		
1. Yes	57	95.0
2. No	2	3.3
3. Don’t Know	1	1.7
How often should one visit a dentist for regular check-up? (choose one only)		
1. Twice an year	44	73.3
2. Once an year	3	5.0
3. Every two years	0	0
4. Only when there is a dental problem	13	21.7
What is your main source of oral health information? (choose one only)		
1. Media (television, radio, newspapers)	27	45.0
2. Your dentist	30	50.0
3. Your relatives/friends	2	3.3
4. Your institution	1	1.7

**Table-II:** Response to questions regarding oral hygiene

*Question*	*Number*	*%*
How often one should brush teeth?		
1. Once a day	3	5.0
2. Twice a day	15	25.0
3. Three times daily	27	45.0
4. After every meal	15	25.0
5. Once or twice a week is enough	0	0
What you mostly use for tooth cleaning? (Choose one only)		
1. Toothbrush	43	71.7
2. Miswak	2	3.3
3. Toothbrush and miswak	15	25.0
If you use toothbrush; do you use toothpaste with brush?		
1. Yes	58	100
2. No	0	0

**Table-III:** Response to questions about fluoride, and dental caries

*Question*	*Number*	*%*
Have you heard of fluoride?		
1. Yes	59	98.3
2. No	1	1.7
What is the main benefit of fluoride? (Choose one only)		
1. It whitens the teeth	15	25.0
2. Protect teeth from dental caries	43	71.7
3. Provide protection from gum diseases	1	1.7
4. I don’t know	1	1.7
What are the various sources of fluoride? (Choose as many as you like)		
1. Drinking water	12	20.0
2. Tooth paste	56	93.3
3. Direct application by dentist	31	51.7
4. I don’t know	2	3.3
Which of the following food group mainly causes tooth decay? (Choose one only)		
1. Too much meats	1	1.7
2. Sugary Foods	59	98.3
3. Too much oily food	0	0
4. Fresh fruits and vegetables	0	0
Which of the following drinks can have harmful effects on the teeth?		
1. Soft drinks such as Coke or Pepsi	59	98.3
2. Flavored fizzy drinks	36	60.0
3. Sweetened/flavored milks	26	43.3
4. Bottled/Canned juices	18	30.0
5. Fresh milk	4	6.7
6. Fresh juices	2	3.3
What should you do when you feel a cavity starting in your tooth?		
1. Wait till it is large enough to be filled	3	5.0
2. Wait till there is some pain in that tooth	8	13.3
3. Visit you medical practitioner	5	8.3
4. Visit a dentist immediately	44	73.3

**Table-IV:** Response to questions about gingival health

*Question*	*Number*	*%*
Blood on your tooth brush during brushing may be a sign of:		
1. Gum disease	57	95.0
2. Tooth decay	2	3.3
3. I don’t know	1	1.7
What should you do when you see blood on your toothbrush?		
1. Stop brushing	1	1.7
2. Stop brushing the area that is bleeding	4	6.7
3. Continue brushing	22	36.7
4. See a dentist	33	55.0
5. I don’t know what to do	0	0

The workers demonstrated adequate knowledge about blood on toothbrush during brushing, though differing in opinion about the action to be taken on seeing the blood. A study in medical nurses in Lesotho demonstrated adequate knowledge of periodontal disease.^15^ Another study of nursing home staff in Singapore also showed good knowledge of periodontal disease.^22^

The study has provided useful information about oral health knowledge and practices in the special health care workers in a Disabled Children’s Center. Although, oral health knowledge and practices among the workers could generally be labeled as satisfactory, some weak areas (such as trend towards symptom-oriented utilization of oral health care and lack of knowledge about fluoridated water) were identified. There is a need to enhance the workers’ knowledge in these areas. Parents/guardians of the special children also refer to these workers for information on oral health care at home. The workers need to be provided educational programs on preventive interventions in special children, so that they can become more effective in improving and maintaining optimum oral health in special health children under their care. The present study is first of its kind in English language health literature. It is likely to generate interest and encourage researchers to conduct similar studies in other parts of the world, consequently resulting in better oral health care of special children.

## CONCLUSION

The special health care workers in the studied disabled children’s center generally have satisfactory oral health knowledge and practices. However, they need more information in areas such as importance of regular check-up visits, use of fluoridated water, and need to visit a dentist immediately in case of any symptoms such as bleeding gums. There is a need of educational programs for special health care workers on oral health prevention.
